# Unique benefits of evenamide on social functioning and life engagement in patients with TRS, confirmed in a placebo-controlled study in inadequate responders

**DOI:** 10.1192/j.eurpsy.2026.12198

**Published:** 2026-07-31

**Authors:** R. Anand, A. Turolla, G. Chinellato, F. Sansi, R. Hartman

**Affiliations:** 1 APC, St.Moritz, Switzerland; 2 Newron Pharmaceuticals SpA, Bresso, Italy; 3 NeurWrite, Morristown, United States

## Abstract

**Introduction:**

Currently available antipsychotics (APs) that modulate dopamine/serotonin have demonstrated benefit on positive symptoms; however, improvement of negative symptoms, that are mediated by hippocampal mechanisms, remains elusive. Recently published effects of evenamide in the MAM neurodevelopmental model indicated that evenamide normalizes excessive glutamatergic activity in the hippocampus, the site of dysfunction in schizophrenia, and downregulates hyperdopaminergic activity in the ventral tegmental area (VTA), thereby reversing schizophrenia-associated cognitive and social dysfunctions (Uliana et al, *NPP* 2025). Evenamide has already demonstrated benefits on positive symptoms and disease severity in patients with treatment-resistant schizophrenia (TRS) and inadequate response (Anand et al, *IJNP* 2025; Anand et al, *Neuropharm* 2025). These findings led to an analysis to determine whether glutamate modulation by evenamide improves negative symptoms including socialization.

**Objectives:**

To present *post-hoc* analyses of data from Study 014/015 and 008A, to demonstrate the clinically meaningful benefits of evenamide add-on treatment in patients with schizophrenia.

**Methods:**

Study 008A, a phase 2/3, 4-week, international, randomized, double-blind, placebo-controlled trial, evaluated the efficacy and safety of evenamide 30 mg bid as add-on in patients with inadequately controlled schizophrenia (PANSS 70-85; CGI-S 4-6) despite stable treatment with an atypical AP (including clozapine) at a therapeutic dose for an adequate period (compliance confirmed through plasma levels before randomization). Study 014/015, a phase 2, 1-year, randomized, open-label, rater-blinded, international study, assessed the long-term efficacy and safety of three fixed doses of evenamide (7.5/15/30 mg bid) as add-on to an AP (excluding clozapine) in patients with TRS (PANSS 70-90; CGI-S 4-6).

**Results:**

Post-hoc analyses demonstrated that evenamide was associated with an increasing improvement in social functioning and life engagement. This effect was noted both in a long-term observational study in patients with TRS and in a placebo-controlled study in patients with inadequate response to SGAs in which the improvement was significantly greater compared to standard of care. These benefits together with the previously demonstrated improvement in the PANSS total score, responder rate, CGI-S/C, indicate that glutamate modulation is a powerful mechanism that can produce clinically significant benefits across positive and negative symptoms that appear to increase over time.

**Conclusions:**

Evenamide, by modulating aberrant glutamatergic activity, uniquely produces clinically meaningful benefits and provides a new therapeutic opportunity for patients with TRS and inadequate response to existing treatments.

**Disclosure of Interest:**

R. Anand Consultant of: Newron Pharmaceuticals SpA, A. Turolla Employee of: Newron Pharmaceuticals SpA, G. Chinellato Employee of: Newron Pharmaceuticals SpA, F. Sansi Employee of: Newron Pharmaceuticals SpA, R. Hartman Consultant of: Newron Pharmaceuticals SpA

